# Understanding decision making in security operations centres: building the case for cyber deception technology

**DOI:** 10.3389/fpsyg.2023.1165705

**Published:** 2023-05-23

**Authors:** Andrew Reeves, Debi Ashenden

**Affiliations:** Defence & Security Institute, University of Adelaide, Adelaide, SA, Australia

**Keywords:** cyber security, security operation center, deception, active defence, naturalistic decision making, literature review, thematic analysis

## Abstract

**Introduction:**

A Security Operations Centre (SOC) is a command centre where analysts monitor network activity, analyse alerts, investigate potential threats, and respond to incidents. By analysing data activities around the clock, SOC teams are crucial in ensuring the prompt detection and response to security incidents. SOC analysts work under considerable pressure to triage and respond to alerts in very short time frames. Cyber deception technology offers the promise of buying SOC analysts more time to respond by wasting the resources and time of attackers, yet such technology remains underutilised.

**Method:**

We carried out a series of interviews with experts to uncover the barriers which prevent the effective implementation of cyber deception in SOCs.

**Results:**

By using thematic analysis on the data, it was clear that while cyber deception technology is promising it is hindered by a lack of use cases, limited empirical research that demonstrates the efficacy of the technology, hesitancy to embrace a more active form of cyber defence, issues surrounding the over promising of results by off-the-shelf vendors, and an aversion to interrupting the decision-making processes of SOC analysts.

**Discussion:**

Taking this last point about the decision-making processes of SOC analysts we make the case that naturalistic decision making (NDM) would help us better understand how SOC analysts make decisions and how cyber deception technology could be used to best effect.

## 1. Introduction

A Security Operations Centre (SOC) is a centralised location where a cyber security team monitors, detects, and responds to cyber security incidents. Many organisations operate a SOC as a key component of their sustainability and cyber security strategies (Schinagl et al., 2015). SOCs use various technologies and processes to deter IT infrastructure misuse, and to prevent and detect cyber threats and attacks, security breaches, and online abuse. Once detected, SOCs have responsibility to respond to cyber incidents. Consequently, the role of a SOC analyst is cognitively demanding (Alahmadi et al., 2022). We use the title ‘SOC Analyst’ to capture the variety of role functions within the SOC team including analysts, operators, and subject matter experts. These team members operate with the intent to expertly respond to identified vulnerabilities and to detect attempts to infiltrate an organisation’s information systems (Alahmadi et al., 2022). They are required to practice perpetual vigilance and continuously monitor security alerts, endpoints, sensors, IT infrastructure, applications, and services, for signs of intrusion or other IT abuse behaviour (Onwubiko, 2015). Their role is also emotionally demanding as they operate in a high-stakes, high-pressure environment where their decisions have critical impacts on the operation of the organisation. Their decisions are unavoidably emotion-laden, regardless of whether they identify signs of attack. That is, their decision to classify a pattern of traffic as malicious means that certain systems must be investigated, quarantined, or even brought offline. As such, the SOC role inherently pressures the analysts to ensure that they minimise the number of false positives. However, false negatives can be equally devastating by enabling cyber incidents. Together, these considerations result in SOC analysts describing their roles as ‘thankless’ (Sundaramurthy et al., 2015).

What is clear is that the SOC role frequently requires analysts to make quick and accurate decisions while under pressure and with only limited access to all relevant information. Despite these pressures, many experienced SOC analysts considerably outperform expectations in their ability to make quick and effective decisions in uncertain conditions (Cho et al., 2020). Their tacit knowledge is often unrecorded and difficult to efficiently communicate to others but is nonetheless highly valuable. Consequently, many SOC managers and other leadership figures are hesitant to make changes that may interrupt these implicit sense-making events (Agyepong et al., 2020).

Research also indicates, however, that erroneous decisions may be made by SOC analysts under pressure (Schinagl et al., 2015). For example, in 2013, attackers managed to breach the defence systems of Target, despite an internal SOC detecting the initial infiltration (Plachkinova and Maurer, 2018). In this instance, no action was taken following the detection which allowed significant losses to occur. This event sparked research interest from Kokulu et al. (2019) who quickly identified that there is a lack of understanding about the way SOC analysts make decisions. Many contemporary attempts to maximise the efficacy of SOCs are not informed by decision-making theory, nor do they view the SOC analyst as an individual who is affected by subjective factors such as stress and fatigue (Dalal et al., 2022). Instead, attempts to maximise efficiency often derive from group or organisation-level methods to improve SOC functioning, via interventions to governance structure, interconnectivity between business units, and the delegated authority of the SOC (e.g., Shahjee and Ware, 2022). While these approaches are valuable, without a firm understanding of the cognitive and emotional nature of the SOC role it is difficult to ensure that analysts are best equipped to make effective decisions.

Cyber deception technology could offer a way to buy more time for SOC analysts to make decisions about alerts and how to best respond to them, with the potential added benefit of decreasing erroneous decisions. Cyber deception technology is increasingly becoming part of an active defence toolkit as it has the potential to increase uncertainty and fear in attackers such that they make mistakes, waste resources and leak information about their tools, tactics and procedures (TTPs). Industry vendors are marketing cyber deception technology as a way for organisations to get the upper hand on the attacker, and to take a more proactive approach to building their network resilience (SentinelOne, 2022). To maximise the utility of using cyber deception technology within SOCs, however, there needs to be an evidence base for ensuring that such technology will improve and not hinder how SOC analysts make decisions. To build this evidence base we need first to understand how SOC analysts currently make decisions when an attacker alert is triggered. Doing so will help inform our understanding of how to use cyber deception based on an understanding of both the cognitive and affective components of the SOC environment.

This research reports on the first stage of a project that takes a human-centred approach to understand the role of the SOC analyst and their decision-making processes as an initial step in building an evidence base for how SOCs might use cyber deception technology effectively. To frame our research approach, we leverage existing literature on cyber deception as well as human decision making under pressure and uncertainty. We focus on the use of Naturalistic Decision Making (NDM) as an ideal method for capturing tacit knowledge about decision making in SOCs.

## 2. Literature review

Keeping step with the growing cyber deception industry, researchers have begun to direct attention towards uncovering the factors that lead to effective development and deployment of cyber deception. Without understanding the link between decision making and behaviour in both the network defender and the attacker, however, it is difficult to build an evidence base that demonstrates the benefits of using cyber deception technology. For example, it is currently unclear whether cyber deception principles may cause SOC analyst confusion and increase the likelihood of events like that experienced by Target in 2013. Alternatively, the low false positive rate promised by cyber deception vendors may dramatically improve the ability of SOC analysts to make decisions with confidence. Ashenden et al. (2021) point out that to date most cyber deception research tends to build from a computer science perspective where the scope is often truncated to misdirecting an attacker on a network rather than impacting decision making and behaviour (Cranford et al., 2020; Shi et al., 2020; Sajid et al., 2021). The only other significant research that has linked cyber deception and decision making is the Tularosa study by Ferguson-Walter et al. (2018). This study leveraged deception techniques to examine both the attacker and defender’s (e.g., SOC analysts’) cognitive processes. Ashenden et al. (2021) build on this research and explicitly use a definition of deception that links deception, decision making and behaviour [Ashenden et al. (2021)] using this as a foundation from which to explore the potential of cyber deception using design thinking as a method.

While a review of the literature identifies a lack of research which specifically targets SOC analyst decision making in relation to the deployment of cyber deception. There is, however, a fledgling research base which investigates the subjective experiences of SOC analysts that we can leverage to frame our research questions. In Sundaramurthy et al. (2015) took a grounded theory approach to understand the experiences of SOCs in managing themselves under pressure at work. They found that SOCs can contain unwittingly vicious cycles that impact SOC analyst morale, leading to low retention and high rates of burnout. Moreover, the frequently challenging decision-making environment inherent in a SOC has deleterious effects on SOC analyst’s mental health and decision-making resilience over time, leading to far from optimal SOC performance.

Similarly, Sundaramurthy et al. (2014) employed an anthropological approach by training a series of computer science students in anthropological methods and embedding them as security analysts in three different SOCs. They found evidence that, far from the parallel process expected in a normative decision, many SOC analysts follow “hunch feelings” (p. 47), which are commonly accurate despite their intuitive and non-analytical nature. The authors assert that incident response has “become so sophisticated and expertise driven that understanding the process is nearly impossible without doing the job.” They note that the incident response job is highly dynamic, as responders attempt to understand constantly evolving threats. One SOC manager indicates that:

“The tasks performed in this job are sophisticated, but there is no manual or textbook to explain them. Even an experienced analyst may find it hard to explain exactly how they discover connections in an investigation” (Sundaramurthy et al., 2014, p. 55).

It appears that this manager is describing skilled intuitions, a type of decision-making process characterised as being quick and heuristic, and yet which are often highly effective and adaptive (Kahneman et al., 1982). Decisions of this type (known as Type 1) contrast Type 2 decisions which are slower, more deliberative, often more ‘rational’, but less effective in some circumstances (Kahneman and Klein, 2009). The distinction between Type 1 and Type 2 decision-making processes is often seen as an overly simplistic model of decision making but it can be a useful way to explore the decision making occurring in complex socio-technical systems (Reeves et al., 2021). In their paper, Cho et al. (2020) explore the application of tacit knowledge in SOCs. Tacit knowledge, often defined as the knowledge that is difficult to transfer or articulate, has been shown to be beneficial in expediting problem-solving procedures in medical emergency responses. However, there is a lack of understanding of its application in IT and socio-technical management, specifically in SOCs. Using Root Cause Analysis (RCA), Cho et al. (2020) were able to identify the procedural elements of tacit knowledge in several scenarios and argue that this research lays the groundwork for tacit knowledge management in SOCs, providing a better understanding of how it is used and how it can be effectively transferred within these organisations. A promising next step would be to apply NDM methods to uncover this tactic knowledge as informed by an earlier RCA.

Further exploring the tacit aspects of decision making occurring within contemporary SOCs, Happa et al. (2021) evaluate the effectiveness of a decision support tool for SOC analysts. The authors conduct a user study to assess the tool’s ability to support analysts in decision-making, triage, and prioritization tasks. The results indicate that the tool can assist analysts in making more informed and efficient decisions. The capture and effective transfer of tacit knowledge and Type 1 decision making performed by SOC analysts is an ongoing challenge for SOC organisations, noting the currently high turnover rate for SOCs.

Studies that explore SOC technical systems and SIEMs often unwittingly uncover the subjective experiences of the SOC analyst role. Therefore, we can look to these studies to complement the limited number of studies that directly explore SOC analyst experiences at work. For example, Feng et al. (2017) identified that the number of alerts that a SOC must investigate is overwhelming, with a majority of flags being false positives. They argue that it is unrealistic to assume that SOC analysts will treat all flags equally, and they will start to fall-back on quick rules of thumb or Type 1 processes to action large batches of flags.

Similarly, Alahmadi et al. (2022) investigated the issue of false positive alerts produced by security tools and the perception of their quality among SOC analysts. Through an online survey of 20 analysts and interviews with 21 analysts, they found that most alarms are attributed to benign triggers or were explained by legitimate behaviour in the organization’s environment. Analysts reported that this high rate of false positive alerts made their role highly stressful, as each alert required investigation or risk reprimand should they misattribute an alert as being benign. Thus, it is critical that any new tool deployed into a SOC does not increase false-positive rate or subjective cognitive load on analysts.

Noting the lack of current knowledge regarding best-practice methods of operating a SOC, a literature review by Vielberth et al. (2020) attempted to uncover the state-of-the-art as seen in research. They identify a series of people-related challenges currently faced by SOCs that limit their efficacy in practice. Specifically, they identify the constant challenge of maintaining accurate decision making when SOC analysts must (1) perform monotonous and demotivating tasks, (2) integrate knowledge across domains, and (3) collaborate and transfer knowledge with other experts in an efficient manner. Finally, they acknowledge the vast difficulty of achieving the three identified tasks when analysts must cope with learning new systems as they are introduced to the SOC, when there is a lack of skilled staff available to SOCs, and given the ongoing retention problem (Vielberth et al., 2020).

We believe that these pressures together highlight the need to thoroughly examine any new technique or technology before implementing into existing SOC procedures to ensure that it is optimised and avoids being an added burden or distraction. Consequently, we believe that the current research program is well timed to address these pressing research gaps. Overall, these papers highlight the multifaceted nature of SOCs and the need for a solid understanding of the decision making occurring within SOCs by SOC analysts to enhance the efficacy of implementing cyber deception techniques.

## 3. Current study

The above literature review provided context to frame our research questions when interviewing experts on the challenges facing contemporary SOCs. Studies such as Alahmadi et al. (2022) and Onwubiko and Ouazzane (2022) have shown that the high rate of false positive alarms produced by security tools is an operational challenge for SOC analysts, and it is unclear how cyber deception would influence this false positive rate. Additionally, research such as Cho et al. (2020) has shown that tacit knowledge plays a crucial role in the decision-making processes of SOC analysts, and that simulations and physical proximity with analysts and vendors can facilitate the transfer of this knowledge. However, this tacit knowledge is often uncaptured by industry and academia alike, and as such it is not clear how to best integrate cyber deception into well-established SOCs where analysts likely use tacit knowledge and highly valuable habituations to effectively action their role. Interrupting these tacit processes with an ill-considered deployment of cyber deception principles may impact SOC efficacy.

As we have seen, there are few research projects that look at real-world behaviour and cyber deception or take a naturalistic decision-making perspective. The few studies that have been carried out include Gutzwiller et al. (2019) who used a ‘think aloud’ technique to better understand the impact of cognitive biases on deception. In addition, research by Shade et al. (2020) highlighted the importance of emotional experiences and the impact of deception.

We believe that NDM methods are well placed to identify success and risk factors for cyber deception deployment. NDM offers us a way to better understand the decision-making processes of SOC analysts in a real-world setting by focusing on the value of expert intuition. Whereas the heuristics and biases approach focuses on experiments that demonstrate how intuitive judgement is often flawed, the NDM approach looks at instances where intuitive judgement by experts is successful. The early work on NDM came from a study of firemen (Klein et al., 2010) who make decisions ‘under conditions of uncertainty and time pressure and that preclude any orderly effort to generate and evaluate sets of options’. The main difference noted in the heuristic and biases approach and the NDM approach is around their starting point (Kahneman and Klein, 2009). Whereas researchers using heuristics and biases look for opportunities to improve decision making by imposing formal models and rules, often via algorithms, NDM researchers are sceptical about formal rules in ‘complex contexts’ (Kahneman and Klein, 2009). Building on this, and as a first step in understanding decision making in SOCs, we designed a qualitative study that involved a small set of expert interviews to scope where NDM interviews with SOC analysts should focus to better understand the potential of cyber deception technology in SOCs.

The study described in this paper explores the following research question:

1. What decision making factors do subject matter experts see as important considerations when deploying cyber deception for use by SOCs?

## 4. Method

### 4.1. Design

We utilised a qualitative research design consisting of semi-structured interviews with Subject Matter Experts (SMEs) from industry and academia. The interviews comprised a set of open-ended questions. These questions aimed to elicit their perceptions on: (a) what factors impact the success or failure of cyber deception deployment, (b) how SOC analyst decision making relates to these factors, and (c) how cyber deception may have both possible positive and negative implications for the SOC analyst.

To continue the discussion, the interview guide contained questions relating to possible legal issues related to cyber deception deployment, what level of abstraction is best for cyber deception tools, and how the progress of AI/ML technology in the field may alter these factors. While this may appear to move away from the intended goal of solely capturing perceptions of SOC analyst decision making in the context of cyber deception deployment, the interviewer was careful to guide the conversation and focus on how these factors may impact the current state of SOC analyst perceptions of cyber deception principles. Appendix A contains the interview guide used for all interviews.

### 4.2. Data collection

The full interview lasted an average of 47 min (min: 27, max: 57). Ethical approval for the conduct of this research was provided by the University of Adelaide, Human Research Ethics Committee. The participants represented a variety of different career backgrounds, organisations, and industries. We believe this provided a solid breadth of perspectives in our sample.

### 4.3. Participants

Four semi-structured interviews were conducted during January–February 2023. Recruitment was performed by email invitations using the researchers’ professional connections. All participants were required to be 18 years of age and to have expertise in the area of cyber deception. 75% of the sample was male (3/4 participants), and the participants worked in a variety of industry contexts and specialisations. Specifically, our sample included one participant working in a Defence context, one in a Defence Industry organisation, one in a Think Tank, and one from Industry Vendors.

The intention in this study was not to interview a representative sample, but to examine rare and particularly informative subject matter experts. Therefore, we recruited a breadth of participants to gain diverse perspectives across industry and defence contexts with participants from both the United Kingdom and Australia. Given that cyber deception remains a niche focus of only a limited number of cyber security professionals, our decision to recruit only those with demonstrated subject matter expertise necessarily led to a small but informative sample.

### 4.4. Data analysis

We utilised a thematic analysis approach accelerated by the Leximancer cloud software platform. The lead researcher analysed the notes taken during the interviews for themes of relevance to the research questions. To accompany this analysis, a datafile consisting of all anonymised interview transcripts was analysed using the Leximancer software platform (Leximancer.com). This produced a list of 58 nodes or concepts in Leximancer which are categorised into six main themes. The notes and the Leximancer codes together form the ‘dataset’. After this initial analysis, we explored the coded data beneath the six themes identified by Leximancer to generate informative titles and description of the themes. Themes were then renamed, merged, or split until the final set of themes provided a useful summary of the dataset (Braun and Clarke, 2006). We found the Leximancer themes broadly agreed with the themes identified in the researcher notes. Leximancer was used to produce a concept map of the identified themes and a Venn-diagram to indicate relative interdependence of the themes. Greater connection of Venn circles via nodes indicates a greater degree of interconnectivity between the themes as observed in the dataset.

Frequency statistics were calculated to enable more detailed descriptions of the concepts and themes. We consider this approach appropriate as it allows statements about frequency, such as “many” or “often,” to have a more precise inference, as recommended by Maxwell (2010). It is worth noting that such statistics may appear to imply undue generalisability of the data (Patton, 1990). In keeping with previous literature, our quasi-statistics consist of simple frequency, count, and percentage data which we use solely for the purposes of pattern recognition, frequency, and group comparison (Maxwell, 2010).

## 5. Findings

**Figure 1:**
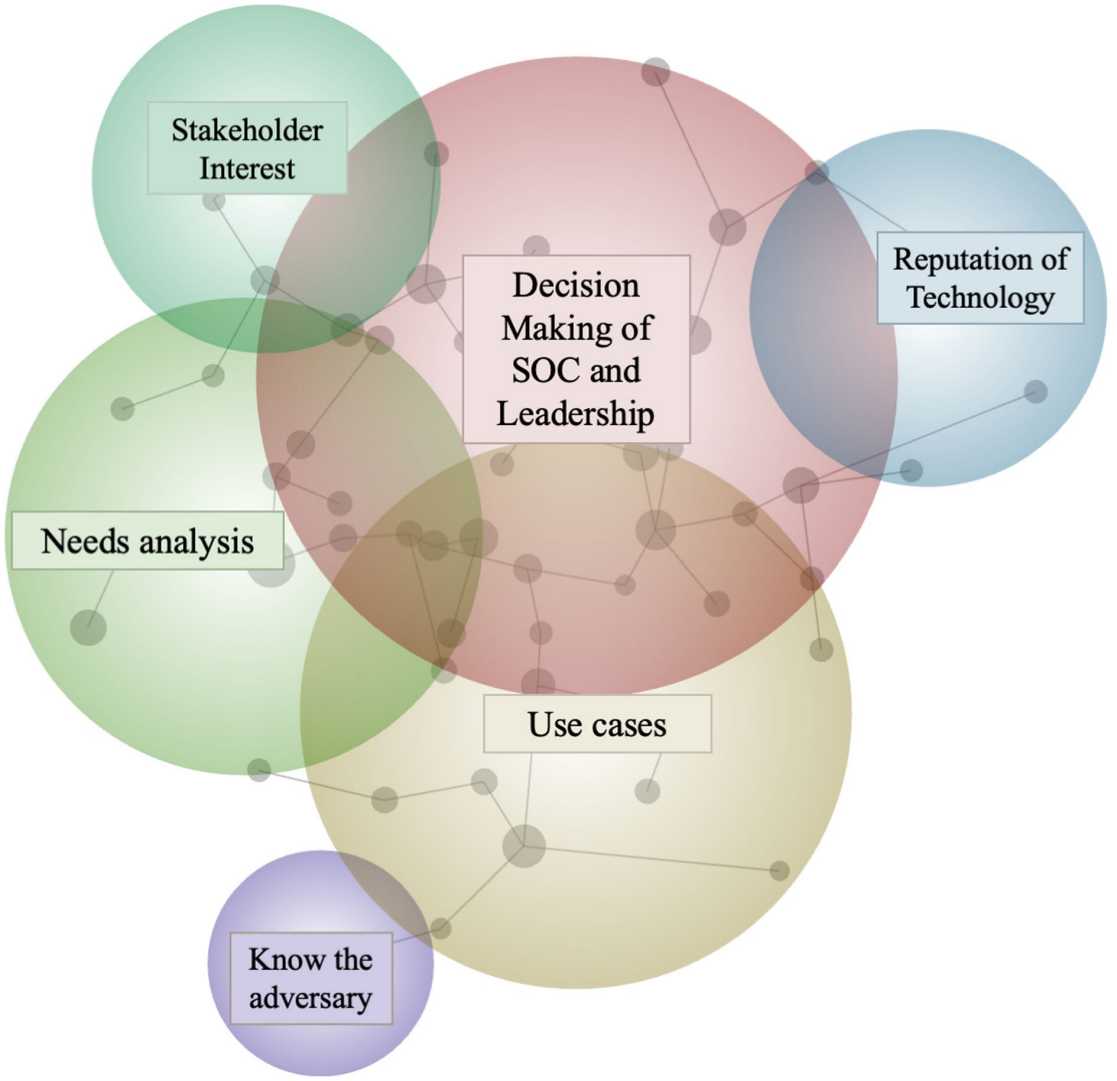
A concept map presenting the six identified themes in the dataset.

Manual coding of the dataset and inspection of the Leximancer output identified six main themes as detailed in the concept map diagram presented in Figure 1.

The six main themes encompassed 58 underlying subthemes (or ‘concepts’ in Leximancer terminology). Each theme’s subthemes were investigated to generate an informative theme title. For example, Leximancer provided Theme 2 with the automated title of “*Use*.” Subthemes included “example,” “sector,” and “understand,” leading the researcher to rename Theme 2 to “*Use cases needed*.”

The overlap between themes connected by nodes in Leximancer outputs (Figure 1) is meaningful (Harwood et al., 2015). The overlap between circles in the Leximancer output reflects the co-occurrence of different concepts in the text and can provide valuable insights into how the concepts are related and how they should be understood.

In the context of our dataset, the overlap between themes can also help us understand the decision-making processes of SOC agents and other decision makers. Specifically, Figure 1 indicates a connection between circles representing “use cases” and “know the adversary.” Consequently, we can infer that experts in our dataset discussed the importance of having a good understanding of the adversary when making decisions about use cases.

Moreover, the overlap between the “decision making” theme and others of “needs analyses,” “stakeholder interests,” and “reputation of the technology,” can give us further insight into the factors that influence decision making. For example, the moderate degree of overlap between the themes *Decision Making* and *Use Cases* indicate that participants’ discussion of these topics tended to intersect, suggesting that leadership teams and SOC analysts value clear and established use cases when making their decision to implement deception technologies in their networks. The overlap between these themes implies decision makers in our dataset consider a variety of factors when making decisions, including the needs of stakeholders, the reputation of the technology, and a thorough analysis of the situation. Table 1 presents these themes and the frequency they were identified in the dataset.

**Table 1:** Coding scheme and main themes.

Main themes	Brief description	Instances found in the dataset	Percentage of total (%)
(1) Decision making (Of both SOC analysts and Leadership)	SOC analysts and leadership figures make complex decisions under considerable uncertainty and pressure. Individual experiences, context, and personal factors will impact the outcome of decisions regarding cyber deception.	491	42.8
(2) Needs analysis	Cyber deception often struggles to demonstrate efficacy due to off-the-shelf implementations pushed by some vendors. Greater value will be added if cyber deception is implemented via a carefully managed and strategic approach that is informed by an understanding of the adversary.	239	20.8
(3) Use cases	Use cases are required for organisations to readily adopt deception technology, yet they are infrequent in the industry and often limited in scope and detail.	217	18.9
(4) Stakeholder interest	Many organisational decision makers and other key players have limited appetite for cyber deception as it appears too advanced for their current needs. There is a common view that many organisations are simply focusing on ‘the basics’.	127	11.1
(5) Reputation of deception technology	There is limited publicly available empirical evidence which demonstrates the efficacy of cyber deception, over and above what can be achieved by more traditional means. In addition, some senior figures in organisations have prior experiences where less sophisticated attempts at deception failed to reach the intended goal, and thus they do not pursue further attempts at implementation.	58	5.1
(6) Know the adversary	Organisations can find it challenging to identify their key adversary, and thus they are unsure how to best face their foe. Consequently, it can be hard for organisations to know where deception should fit in their network, and how they would measure success.	16	1.4

### 5.1. Theme 1: decision making of SOC analysts and leadership

Unsurprisingly the most common topic of discussion was the decision-making processes occurring within a SOC. This discussion involved a consideration of the difficulties experienced by SOC analysts who must decide if an alert is a simple false positive or a sign of a larger issue warranting attention. Furthermore, participants discussed the multifaceted nature of decisions occurring at leadership levels as it pertains to SOCs. Specifically, it was commonly reported that a solid understanding of the decisions being made at a Board, Executive, and a local level play a key role in the success of implementing cyber deception within the SOC and the broader organisational networks.

If you're just thinking about [implementing deception technology] from a regulatory, tick-box mindset, then you're in the wrong game. You've got to think about: what we should we be focusing on? If you're not having a constructive conversation with the whole of the organisation’s decision-making bodies, then you're not doing it right (Participant 1, Defence).

Furthermore, participants indicated that the success of cyber deception will significantly rely on the ability of the SOC analysts and leadership to understand the benefits and limitations of cyber deception, and to make decisions that align with the organization’s objectives and priorities. For instance, decision makers must consider the potential risks of implementing cyber deception, such as the possibility of false alarms, or the complexity of managing the technology. They must also weigh the benefits, including improved threat detection, reduced response times, and improved incident management.

That is part of the reason why some organisations might be resistant, because it's such a bigger, different way of doing things. Deception technology can't just be slotted into their existing tech stack. I think the resistance is twofold around that one, because it involves the leap to using your SOC in a different way, and changing your risk appetite, and having a comfortable relationship with legal and policy about where you stand, where you sit, what's possible, what's not possible, what you want to do. But with a limited research body to know how this is actually going to make things better, not worse, it’s seen as a bit of a gamble (Participant 1, Defence).

The mindset of the decision makers is crucial, as they must be able to assess the benefits and limitations of the technology and make informed decisions that align with the organization’s objectives. As such, decision-making skills coupled with training are essential for SOC analysts and leadership figures to ensure the effective implementation and use of cyber deception.

### 5.2. Theme 2: needs analysis.

The majority of participants discussed the need for organisations to take a well-considered and carefully planned approach when implementing cyber deception, and to avoid simply purchasing off-the-shelf tools for installation into their existing technology stack. Reinforcing this attitude, it was commonly discussed that many vendors sell cyber deception as a ‘set-and-forget’ toolkit, rather than as a comprehensive and adaptable solution.

When you're doing deception for deception's sake, there's a limited value and a limited return. When you look at deception as supporting elements of a larger strategy, I think that's where you really start seeing value (Participant 2, Industry Vendor).

If you look at military use of deception in the US, deception was a supporting element of the commander's strategy. It was not the commander's entire strategy. I think a lot of times with deception it's sold as: put this super subatomic honeypot on the blockchain-you know, with all the buzz words–and it's gonna solve all your problems. But if an organisation just goes and buys all these great deception appliances, throws it on their network and walks away, they’re gonna have diminishing returns (Participant 3, Think Tank).

To maximize the benefits of cyber deception, most participants felt that organisations should perform a needs analysis to understand what gap the technology is meant to fill in their existing defence procedures. This will help them identify the specific areas where the technology can be most effectively deployed and will also help them understand how the technology can be integrated into their existing security infrastructure.

We really need to do that planning and analysis, and really asking: What are you trying to accomplish? What is your objective? What is the action that you need the adversary to take? One that is beneficial to you, the defender? What do you need to do to get there? We talk a lot about the ‘See, Think, Do’ model: What do you need your adversary to see? What do you want them to think? What do you ultimately want them to do? (Participant 3, Think Tank).

While purchasing vendor products might be a quick and easy solution, participants emphasised that the implementation of cyber deception requires a thoughtful and strategic approach. Organisations can maximize the potential benefits of cyber deception and better protect themselves from cyber threats by fully understanding what the technology is meant to do and how it can be integrated into their existing infrastructure.

### 5.3. Theme 3: use cases, and related theme 6: knowing the adversary

As shown in Figure 1, the sixth theme: *Knowing the Adversary*, is connected to the first theme: *Decision Making*, via the third theme: *Use Cases*. Therefore, the following section details the participants’ views on the importance of use cases when coming to decisions, and how these use cases should be informed by a solid understanding of the adversary.

Many participants discussed how the effective use of cyber deception in SOCs depends on the creation of innovative and evidence-based use cases. A use case can be considered a specific scenario in which the technology is applied to a particular problem or situation (Chen, 2020). Without a clear use case, participants indicated that SOC leadership may struggle to understand the value of the technology and how to effectively implement it.

If you look at the risk appetite of most organisations, they just want to shut [attacks] down and make them go away because of regulatory drivers or other pressures inside the business. They don’t want to gain deep intelligence or understand of their adversaries. And so they don’t see a use case for some deception technologies (Participant 2, Industry Vendor).

Our interviews uncovered that a key challenge in the development of clear use cases for cyber deception is the need for good knowledge of the attacker or adversary. That is, to understand how the technology can be used to deceive an attacker, it is important to have a good understanding of the attacker’s tactics, techniques, and procedures (TTPs). Without this knowledge, it is difficult to develop a use case that is tailored to the specific attacker and will be effective in deceiving them.

We stood up a honeypot, and we caught half of the attack, which allowed us to use the deception product to ascertain where the vulnerability was that the attacker was exploiting. This allowed the organisation to then mitigate the vulnerability. We are seeing organized crime explore a range of different techniques and so just getting insight into their modus operandi, and getting into their tradecraft, is terribly important from an attack mitigation perspective (Participant 1, Defence).

Unfortunately, research on adversary methods and tactics remains limited. Many organisations do not have the resources or expertise to conduct this type of research, and there is a lack of publicly available information about attackers and their TTPs. As a result, it can be difficult for organisations to develop effective use cases for cyber deception.

There's very little research out there that confirms deception is a worthwhile thing to do. It feels like it's just one more thing to do, and I can point to a bit of research to do with state actors and a few other things. But most of the research in deception space doesn't exist (Participant 1, Defence).

I think we're a long way from where we need to be, in terms of adopting what some would refer to as an adversary engagement. Mentality, for most organisations, especially small to medium size enterprises, they're still struggling to really keep their heads above water on some of the basics. So, deception is still looked at as kind of a more elite technology (Participant 3, Think Tank).

### 5.4. Theme 5: reputation of cyber deception principles

Several participants discussed how SOC leadership figures often have limited appetite to pursue deception in their own networks due to a lack of consensus in the broader industry of the utility of the technology. It was commonly noted by participants that, despite its potential benefits, cyber deception has a mixed reputation within industry and academia alike due to concerns about its effectiveness and potential drawbacks. In particular, it was noted that the low number of alerts produced by well-implemented deception systems can make it difficult to demonstrate the efficacy of the system.

I've watched companies who have tried various things where we will deploy [cyber deception], and then we will role play what it would be like for the organisation to receive an alert, but that is an artificial exercise. They’ve seen how the trick works, and we tell them: trust me when this happens for real, it will be really good. So, you can get an executive who says, look: we haven't had any hits on this, this is not doing anything (Participant 4, Defence Industry).

[Deception technology] actually appears less successful than a system that alerts all the time. I think that's more of a criticism of the way we do metrics than it is the criticism of the technology. It's causing some problems for the vendors to be able to show proof of work and proof of effectiveness, because someone goes and does a proof-of-concept for 6 months, and none of their alerts go off. Well, did none of your alerts go off because nothing was there? Or did none of your alerts go off because something was there, and you missed it? And I think sometimes they do these 6-month POCs [proof of concept] and nothing happens, and the company says: oh, we don't need deception. But on day 6-months-and-one-day something bad happens, and deception would have caught it (Participant 3, Think Tank).

One of the main concerns among SOC leaders is the potential for false positives and false negatives, and our interviews indicated the industry currently has not reached a consensus as to whether cyber deception may increase or reduce false-positive alerts reaching the SOC. These concerns can lead to confusion and uncertainty among SOC leaders, making it difficult for them to make informed decisions about whether to deploy cyber deception. Furthermore, some participants suggested that there are frequent misconceptions about the potential for deception technology to open new vectors of attack for unauthorised access to production systems.

Another misconception by the market is that you are going to increase your risk by deploying deception. It's almost like, let’s build a decoy house, but what if we deliberately going to lead them back to our real house? Which no, we won't. We won’t do that. We'll have a completely different house that does not and cannot be connected to your house. So that's a common misconception, and sometimes people do things to deploy in a way that's risky, and for certain circumstances that an organisation may wish to do that for a specific reason, but generally in the commercial enterprise space, we should never increase exposure of the production environment through the deployment of deception (Participant 4, Defence Industry).

## 6. General discussion and future research

The results of the interviews with experts uncovered six key themes that may present barriers for the implementation of cyber deception technology: a lack of clear and established use cases, the limited amount of empirical research that demonstrates the efficacy of the technology, a general hesitancy by some decision makers to embrace a more active form of defence and security, the overpromising of results by off-the-shelf vendors of deception technology, the difficulty involved in truly understanding the adversary, and a limited knowledge on how effective decisions occur within SOC analyst groups and SOC leadership.

This supports our initial conclusions that a promising next step for research would be to investigate the complex decision making of SOC analysts, leadership, and other relevant organizational decision makers in a real-world context. This research could provide insights into the factors that are critical to understand for a successful deployment of cyber deception and how these factors influence the decision making of the relevant actors. The interviews suggest that adopting a naturalistic approach (Klein, 1993) using the critical decision method or observational methods (Flanagan, 1954; Koleva, 2015) will help us achieve an in-depth understanding of decision making in a SOC. This aligns with the work of Alahmadi et al. (2022), who demonstrated the utility of a qualitative, naturalistic approach to implementing novel tools into existing SOC procedures. Their work highlights the need for research that takes into account the perspectives of SOC analysts and related decision makers in order to properly evaluate the adequacy and implementation of deception tools. These findings also build on the work of Onwubiko and Ouazzane (2022) who argue for greater adoption of deception technologies in order to drive the development of a common and consistent lexicon for incident management, and to aid collaboration and knowledge sharing. However, our findings indicated that for this market penetration to be realistically achieved, the reputation of cyber deception must be improved (Theme 5). To this end, research is needed to better understand how cyber deception can be implemented in SOCs to support its potential benefits and limit any drawbacks. By conducting studies that evaluate the effectiveness of the technology and investigate ways to mitigate potential drawbacks, researchers can help to build trust in the technology and demonstrate its value to SOC leaders. Additionally, by collaborating with SOC leaders and other stakeholders, researchers can gain valuable insights into the challenges that SOC leaders face and develop solutions that are tailored to their needs. Building stakeholder interest in this way can ensure that the research is relevant and useful to the individuals and organisations that will be implementing the solutions.

Furthermore, there is a need for future research to find methods to capture the tacit knowledge of experienced SOC analysts, as the ongoing loss of this information was highlighted by both our literature review and by our expert panel. Following existing knowledge-capture frameworks such as that built by Cho et al. (2020), SOCs and researchers may begin to facilitate tacit knowledge capture, and transfer these skills to less experienced SOC analysts. This may be achieved through use of simulation environments (Cho et al., 2020), critical decision analysis (Flanagan, 1954; Koleva, 2015), and observation (Klein, 1993). Our results indicate that NDM research is well placed to answer many of the questions raised in the interviews and Table 2 summarises the relevance of NDM to addressing some of the barriers identified in the interviews which impact cyber deception deployment.

**Table 2:** The relevance of NDM to exploring the barriers identified in the interviews that impact Cyber Deception deployment.

Barrier (related theme)	Relevance of NDM
(1) We currently do not understand the decision making of SOC analysts and Leadership (Theme 1)	NDM methods can provide insights into how expert analysts and leaders make decisions in real-world settings. By studying their decision-making processes, NDM can help identify and develop best practices and decision-making tools to improve the quality and speed of decisions made by SOC analysts and leadership in regard to incorporating Cyber Deception principles in their networks and systems.
(2) Needs analyses can be challenging and are often not performed (Theme 2)	NDM can help identify the key factors and considerations that should be taken into account during the needs analysis phase, specifically to identify how a cyber deception technique should be best placed within an existing complex set of networks and systems.
(3) An effective needs analysis should be informed by information about the adversary, which is often limited. (Theme 6)	NDM methods can help us understand the methods of the attacker by studying the decision-making processes used by attackers in real-world cyber-attacks and in simulations (e.g., red teaming and penetration testing). By analysing their behaviour, tactics, and techniques, NDM can help identify patterns and insights into how attackers make decisions and adapt to changing situations during an attack. This can include identifying the types of tools and techniques used by attackers, the specific vulnerabilities they target, and the strategies they use to evade detection and maintain persistence. By understanding the decision-making processes of attackers, NDM can help inform the development of more effective defensive strategies, including the implementation of countermeasures and the development of more robust security protocols.

Finally, our findings further highlight the need for researchers to develop methods of demonstrating the efficacy of cyber deception, continuing such projects as the Tularosa Study (Ferguson-Walter et al., 2018). This includes understanding how adversaries may react to deception and developing tools that allow industry to measure the success of deception tactics. Furthermore, understanding the behaviour and tactics of adversaries is crucial in order to effectively design and implement deception strategies.

## 7. Limitations

The study described above has several limitations that need to be acknowledged. Firstly, our sample method may have led to a relatively narrow slice of the industry taking part in our interviews. This limited diversity may limit the study’s ability to capture a range of perspectives and experiences related to implementing cyber deception in SOCs. In addition, our interview was built from initial scoping conversations with key individuals with expertise in cyber deception. Therefore, the interview schedule contains the implicit assumption that cyber deception principles have failed to reach substantial market penetration due to the presence of certain barriers that can be uncovered and addressed. It may be that alternate explanations for the current state of cyber deception utilisation in industry are warranted.

Furthermore, the study focuses exclusively on the barriers to implementing cyber deception in established SOCs. However, there may be other contexts, such as newly created SOCs or organisations that do not have a SOC, where the barriers to implementing cyber deception may differ. Moreover, while the study highlights several barriers to the effective implementation of cyber deception, it leverages the experience of subject matter experts rather than direct empirical observation or experimental methods.

## 8. Conclusion

Cyber deception remains underutilized in industry and defence networks, despite the promise of this approach to provide high fidelity, low volume alerts to otherwise overwhelmed SOCs. This paper reports on interviews with industry experts to uncover the barriers preventing the effective implementation of cyber deception. The results indicate that the market penetration of cyber deception is hindered by a multitude of factors, including: (1) a lack of understanding of how SOCs leverage their existing monitoring software to come to decisions, (2) a lack of clear and established use cases for cyber deception, (3) the limited amount of empirical research that demonstrates the efficacy of the technology, (4) a general hesitancy by some decision makers to embrace a more active form of defence and security, (5) the overpromising of results by off-the-shelf vendors of deception technology, and (6) the difficulty involved in truly understanding the adversary. Consequently, we believe there is a need to understand SOC decision making from an NDM perspective, with a view to inform the positioning and deployment of cyber deception systems, and that this should be a priority for future research programs.

## Data availability statement

The raw data supporting the conclusions of this article will be made available by the authors, without undue reservation.

## Ethics statement

The studies involving human participants were reviewed and approved by University of Adelaide, Human Research Ethics Committee. The patients/participants provided their written informed consent to participate in this study.

## Author contributions

AR and DA contributed to conception and design of the study, and the literature review. AR conducted the interviews, performed the thematic analysis, and wrote the first draft of the manuscript. DA wrote sections of the manuscript. All authors contributed to the article and approved the submitted version.

## Funding

This research was funded by the NGT Cyber Call 2020, and the project was ‘Fusing Behavioural Science and Cyber Deception—Fighting Wars from Inside Machines’. The funder was not involved in the study design, collection, analysis, interpretation of data, the writing of this article, or the decision to submit it for publication.

## Conflict of interest

The authors declare that the research was conducted in the absence of any commercial or financial relationships that could be construed as a potential conflict of interest.

## Publisher’s note

All claims expressed in this article are solely those of the authors and do not necessarily represent those of their affiliated organizations, or those of the publisher, the editors and the reviewers. Any product that may be evaluated in this article, or claim that may be made by its manufacturer, is not guaranteed or endorsed by the publisher.

## Appendix A: interview schedule

*Informed consent gathered and consent form signed.*

*Preamble: As you know, we are currently conducting a research project looking at cyber deception and SOC operations. As part of this, we are keen to better understand where cyber deception could sit within a SOC playbook, according to experts from industry and academia. We also wish to understand how SOC agents may perceive such technologies and how they should be best incorporated into their workflows.*

(Timing: approx. 5 min per question)

1. What do you think prevents or limits the use of cyber deception in the industry?
2. Is there consistent appetite for deception in the industry? Why/why not?
3. What hinders cyber deception deployment?
4. How do you think SOC agents view cyber deception?
5. What are the possible positive and negative impacts of deception technology on SOC Analysts’ decision making?
6. Would this differ for experienced analysts vs. graduates/early career?
7. What tools are best for them? (Fake credentials, fake people, fake files, whole systems?) What level of abstraction? How would they differently affect a SOC’s ability to make decisions?
8. Given these limiting factors, what can we realistically hope to achieve?
9. What gaps exist in cyber deception research?
10. How should cyber deception sit within a SOC playbook?
11. What are the legal issues, if any?

Wrap up, Ask the participant if they have any remaining questions and/or would like to receive information about research progress and findings in the future. Thank them for their time.
